# Vegetable Origin of Porrigo Decalvans

**Published:** 1844-04-20

**Authors:** 


VEGETABLE ORIGIN OF PORRIGO DECALVANS.

Porrigo decalvans is a disease of the skin, usually of the scalp, producing a falling-off of the hair from the parts affected. It is characterised by rounded spots or patches, the surface of which is usually covered with small dry scales, or a white bran-like powder. If this powder be examined with the microscope, it will be found to consist of minute *cryptogamic* plants. The hairs that have fallen off are observed to be encrusted on all sides with these singular growths, and to form a veritable vegetable sheath, which invests them from their point of emergence from the skin to the extent of three or four millimetres. M. Gruby (the discoverer, we believe, of this pathological curiosity) has denominated the fungus by the appellation of *microsporon Andouini*, in compliment to M. Andouin, whose inquiries have done so much to illustrate the nature of the parasitic plants, which infest the tissues of living animals.

This porriginous fungus commences its development on the surface of the hairs, at the distance of one or two *millimetres* from the epidermis; the first sign of its existence being that the tissue of the hair is observed to lose its transparency, in consequence of the formation of excessively minute molecules on its surface.

The altered tissue exhibits the appearance of having distinct fibres, and of cells larger than the fibres of the hairs, elongated and situated parallel to their axes. It is in this part that the *microsporon* is first perceived. By its gradually extending in all directions, the adjoining hairs are quickly affected in a similar manner, and the morbid growth advances until patches of the affected hair fall off, and leave the skin nearly quite bald.

These fungi are developed and multiplied with surprising rapidity, and hence the extension of the diseased patches is sometimes very sudden. The hairs usually break off at the point where they are invested with the vegetable covering. The thickest hairs resist the invasion of the disease the longest. The vegetable nature of *porrigo decalvans* is a fact that speaks for the contagiousness of the disease. How far any of the other varieties of this scalp-affection are traceable to a similar origin has not yet been ascertained. The first step to the cure of a disease being to understand its nature, we may fairly anticipate that the discovery of M. Gruby will have a therapeutic, as well as a physiological, interest.—*Med. Chir. Rev.*, Jan.

*** We have recently been enabled to confirm the observations of M. Gruby. Porrigo decalvans is, in many cases, very intractable, but we have found it yield to local applications, in some instances, of sulphate of iron. Others we have treated successfully by tincture of cantharides, combined with castor oil.—*London Lancet.*

